# Spinal neuron-glial crosstalk and ion channel dysregulation in diabetic neuropathic pain

**DOI:** 10.3389/fimmu.2025.1480534

**Published:** 2025-04-08

**Authors:** Jie Wu, Haijun Hu, Xi Li

**Affiliations:** ^1^ Department of Anesthesiology, The 2^nd^ Affiliated Hospital, Jiangxi Medical College, Nanchang University, Nanchang, Jiangxi, China; ^2^ Key Laboratory of Anesthesiology of Jiangxi Province, Nanchang, Jiangxi, China

**Keywords:** diabetic neuropathic pain, microglia, astrocytes, Schwann cells, ion channels, spinal dorsal horn

## Abstract

Diabetic neuropathic pain (DNP) is one of the most prevalent complications of diabetes, characterized by a high global prevalence and a substantial affected population with limited effective therapeutic options. Although DNP is closely associated with hyperglycemia, an increasing body of research suggests that elevated blood glucose levels are not the sole inducers of DNP. The pathogenesis of DNP is intricate, involving the release of inflammatory mediators, alterations in synaptic plasticity, demyelination of nerve fibers, and ectopic impulse generation, yet the precise mechanisms remain to be elucidated. The spinal dorsal horn coordinates dynamic interactions between peripheral and central pain pathways, wherein dorsal horn neurons, microglia, and astrocytes synergize with Schwann cell-derived signals to process nociceptive information flow. Abnormally activated neurons can alter signal transduction by modifying the local microenvironment, compromising myelin integrity, and diminishing trophic support, leading to neuronal sensitization and an amplifying effect on peripheral pain signals, which in turn triggers neuropathic pain. Ion channels play a pivotal role in signal conduction, with the modulation of sodium, potassium, and calcium channels being particularly crucial for the regulation of pain signals. In light of the rising incidence of diabetes and the current scarcity of effective DNP treatments, a thorough investigation into the interactions between neurons and glial cells, especially the mechanisms of ion channel function in DNP, is imperative for identifying potential drug targets, developing novel therapeutic strategies, and thereby enhancing the prospects for DNP management.

## 1 Introduction

The global rise in the number of diabetes patients has propelled the study of diabetes-related complications to the forefront of medical research. As the third leading cause of mortality worldwide (1), following cancer and cardiovascular diseases, diabetes presents a significant threat to global public health. In 2021, diabetes was diagnosed in 10.5% of the world’s population, with an anticipated increase to a 12.2% prevalence rate by mid-century, affecting an estimated 780 million individuals (2). China, notably, is home to over 110 million adults with diabetes, constituting 24% of the global diabetic population and ranking as the country with the highest number of diabetic patients (3). However, a considerable proportion, approximately one-third to one-half of patients, remain unaware of their condition (4), often receiving a diagnosis only upon the emergence of severe complications or during routine health check-ups. This scenario poses a substantial challenge to the early detection and management of diabetes. Moreover, it amplifies the healthcare burden, particularly in low- and middle-income nations where investment in diabetes care is critically lacking (5).

**Figure 1:**
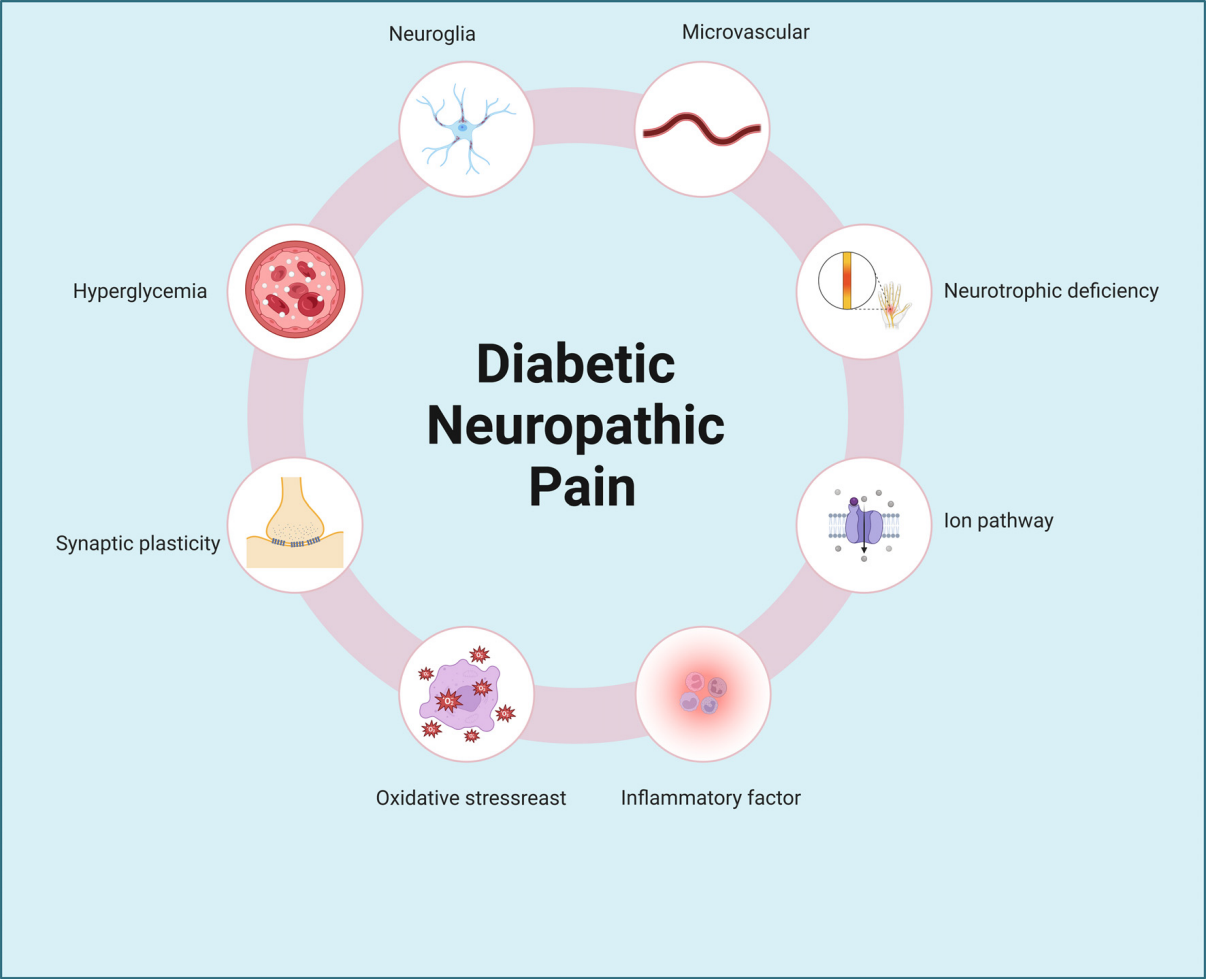
Pathogenic mechanisms of diabetic neuropathic pain. Diabetic neuropathic pain involves complex and multifactorial pathogenic mechanisms, including hyperglycemia, neuroglial activation, synaptic plasticity abnormalities, microvascular dysfunction, neurotrophic deficiency, oxidative stress, ion channel dysregulation, and inflammatory pathways, among others.

Diabetes imposes numerous challenges on daily living, with inadequate blood sugar management leading to detrimental effects on various bodily systems, including the heart, blood vessels, nerves, kidneys, and eyes, potentially resulting in irreversible damage (6–10). Conditions such as diabetic retinopathy, diabetic nephropathy, diabetic cardiomyopathy, and diabetic neuropathic pain exemplify the disease’s role as the fourth leading cause of disability globally, following cancer, cardiovascular diseases, and trauma (1). Notably, diabetic neuropathic pain stands out as a prevalent complication, with nerve damage often manifesting early in the disease’s progression. Consequently, the chronic pain associated with nerve injury significantly diminishes patients’ quality of life. The etiology of neuropathic pain remains elusive, encompassing factors like inflammation, microvascular disease, metabolic stress, and neurotransmitter imbalances (11–14), with many theories yet to achieve scholarly consensus (**Figure 1**). Notably, the dysregulation of ion channel homeostasis in neuronal cells may serve as a critical molecular hub underlying aberrant nociceptive signal transmission.

Spinal dorsal horn is a pivotal center for the relay of peripheral pain signals (15, 16), densely populated with neurons and glial cells that are essential for the propagation of electrical impulses (17). Upon abnormal activation of these cells, Upon abnormal activation of these cells, substantial release of inflammatory mediators and chemokines initiates a pathological cascade. This coordinated dysregulation not only exacerbates oxidative stress but consequently propagates neural conduction disorders, amplifies aberrant neuronal firing, and potentiates maladaptive synaptic plasticity through feedforward mechanisms (18–20). In clinical practice, strategies for managing diabetic neuropathic pain (DNP) commonly include glycemic control (21), neurotrophic support (22), and anti-inflammatory interventions (23). Although these treatments may provide relief in the early stages of DNP, pain and discomfort often persist even when blood glucose levels are well-managed in the later stages. This persistent pain can erode patients’ adherence to treatment and potentially lead to co-morbid psychiatric conditions (24). This article reviews the roles of neurons and supporting cells, such as astrocytes, microglia, and Schwann cells, in DNP. It delves into the cellular mechanisms by which these cells mediate neuropathic pain and examines how alterations in ion channels influence the progression of DNP. The aim is to offer novel perspectives for the mechanistic understanding and therapeutic development of DNP.

## 2 The spinal dorsal horn is a significant participant in the pathophysiology of diabetic neuropathic pain

**Figure 2:**
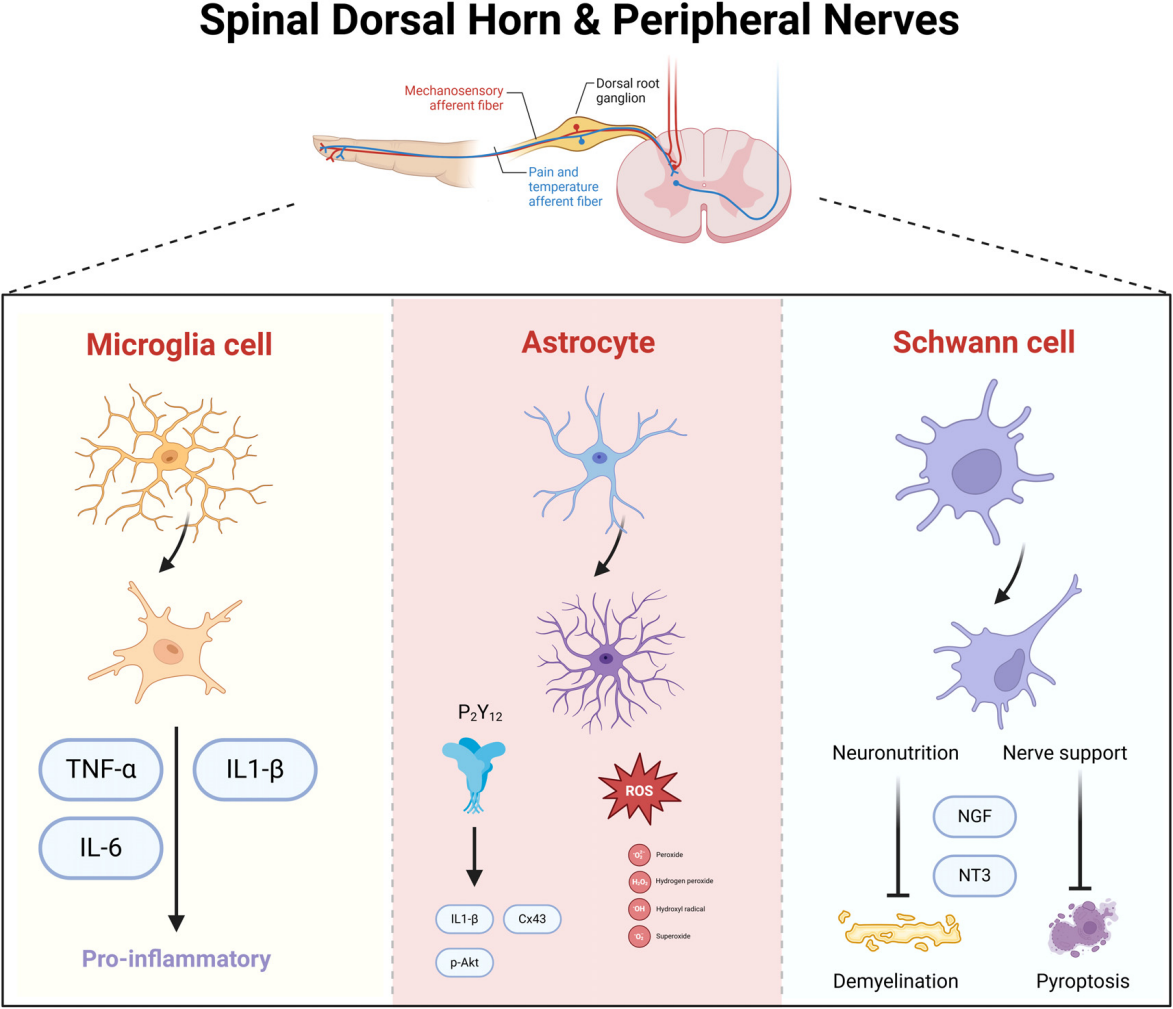
Spinal dorsal horn and spinal nerve neuronal cells in mediating diabetic neuropathic pain. Microglia, astrocytes, and Schwann cells synergistically mediate neuroinflammatory signaling and neuronal hyperexcitability in diabetic neuropathy. Activated microglia release pro-inflammatory cytokines (e.g., TNF-α, IL-1β), amplifying nociceptive transmission. Astrocytes sustain neuroinflammation by propagating cytokine cascades and disrupting glutamate homeostasis, leading to central sensitization. Schwann cells, through peripheral nerve damage, secrete nerve growth factor (NGF), which sensitizes nociceptive neurons and enhances pain signaling to the spinal cord. Neuronal circuits in the spinal dorsal horn integrate these inputs, with neurotrophic factors further promoting synaptic plasticity and chronic pain.

The spinal dorsal horn, situated within the posterior gray matter of the spinal cord, serves as a pivotal relay station for the processing of sensory information from the limbs, with a particular focus on pain. This region is replete with diverse neuronal and glial cell types, integral to the integration and modulation of pain signals (**Figure 2**). The locus coeruleus-norepinephrine system (LC: NE) acts as a significant feedback mechanism, inhibiting the ascending transmission of pain within the spinal cord. Research has demonstrated that dampening the LC: NE pathway can mitigate inflammatory responses and alleviate pain by curbing the activity of astrocytes and microglia (25). In a mouse model of spontaneous encephalomyelitis, Ding et al. (26) observed early astrocyte activation. The anion channel LRRC8A on astrocytes, when activated, facilitates glutamate release, which is intimately linked to neuropathic pain (NP). Recent studies have reaffirmed that the activation of the JAK2/STAT3 signaling pathway drives the conversion of astrocytes and microglia into pro-inflammatory phenotypes, and targeting the upstream regulator IL-6 of this pathway can markedly attenuate DNP (27). The GABAB receptor, abundant in the spinal dorsal horn, exerts a crucial regulatory effect on synaptic transmission and inflammatory processes (28). Stimulation of GABAB receptors can ameliorate DNP by suppressing the TLR4/Myd88/NF-κB signaling cascade (29). While neuronal activation within the spinal dorsal horn can initiate pain via various mechanisms, the temporal dynamics, magnitude, and precise functions of these neurons in DNP exhibit variability. Moreover, the interplay between different cell types amplifies the intricacy of developing targeted therapeutic approaches (**Table 1**).

**Table 1:** Cell-type specific targets and mechanisms in the treatment of diabetic neuropathic pain.

Drug/Intervention	Target	Mechanism	Reference
Electroacupuncture	microglia	Inhibits microglial P2X4 receptor upregulation, alleviating neuroinflammation	(30)
Glucagon-like peptide-1 receptor agonist	microglia	Downregulates microglial NLRP3 expression levels	(31)
Minocycline	microglia	Anterior cingulate cortex microglial activation and upregulation of CXCL12 expression; increased glutamatergic neuron peak potentials	(32)
GS-KG9	microglia	Inhibits microglial activation in the L4 dorsal horn and thalamic VPL regions	(33)
Koumine	microglia	Inhibits M1 microglia polarization and activation of the Notch-RBP-Jκ signaling pathway	(34)
Ginger	microglia	Expression of CD11b gene in microglia/macrophages, improving gut microbiota imbalance	(35)
Teneligliptin	microglia	Nrf2 and HO-1 as well as inhibiting dorsal horn microglial activation	(36)
Bullatine A	microglia	Stimulates spinal microglial enkephalin A expression	(37)
Ammoxetine	microglia	Reduces microglial activity, pro-inflammatory cytokine accumulation, and p38 and c-Jun N-terminal kinase (JNK) activity in DNP rats’ spinal cord	(38)
Achaete-scute homolog 1, LIM homeobox protein 6	microglia	Inhibits microglial activation and pro-inflammatory factors TNF-α and IL-1β as well as p38, c-Jun N-terminal kinase, and NF-κB activation, increases IL-4, IL-10, and IL-13 anti-inflammatory factors levels	(39)
Minocycline hydrochloride	microglia	Inhibits microglial activation and upregulation of XCL1 and XCR1 expression	(40)
Fluorocitrate, neurotropin	astrocytes	Inhibits activation of astrocytes in the periaqueductal gray matter	(41)
Koumine	astrocytes	Inhibits astrocytic activation and reduces inflammation response	(42)
/	astrocytes	Astrocyte activation in the motor cortex causes excitatory neuron activation	(43)
/	astrocytes/microglia	Glial cell activation promotes TNF-α and IL-1β level increase	(44)
/	astrocytes	Increased number and soma size of astrocytes in the ventrolateral periaqueductal gray matter (vlPAG)	(45)
Curcumin	astrocytes/neuron	Decreases pJNK expression levels in DRG astrocytes and neurons	(46)
Dihydromyricetin	astrocytes	Decreases astrocytic P2X7 receptor upregulation, inhibits inflammatory factor release	(47)
MiR-133a-3p	schwann cells	Upregulates VEGFR-2, p38α MAPK, TRAF-6, and PIAS3 expression, and downregulates NFκB p50 and MKP3 expression	(48)
Apocynin	schwann cells	Prevents Schwann cell degeneration and loss, alleviates oxidative stress response	(49)
Ro5-4864	schwann cells	Antioxidant stress, promotes autophagy, improves schwann cell function, and promotes myelin regeneration	(50)
Human tonsil-derived mesenchymal stem cells	schwann cells	Promotes myelin regeneration	(51)

### 2.1 Microglia are actively involved in the regulatory mechanisms of diabetic neuropathic pain

Microglia, as the primary immune cells of the central nervous system (CNS), are pivotal in maintaining CNS homeostasis, facilitating post-injury repair, modulating immune responses, and participating in neuroinflammatory processes (52, 53). Emerging evidence highlights the pivotal role of microglial activation in mediating pathophysiological processes underlying diabetes mellitus and its complications. Under diabetic conditions, elevated glucose levels are known to drive microglial polarization from the anti-inflammatory M2 phenotype to the pro-inflammatory M1 phenotype, thereby augmenting systemic inflammatory responses, and Levetiracetam was utilized to suppress microglial activation, which consequently reduced the secretion of pro-inflammatory cytokines such as TNF-α, IL-1β, and IL-6, and ameliorated cognitive deficits in a murine model (54). Similar observations regarding microglial activation and functional alterations have been reported in diabetic retinopathy (55). Microglia’s role in pain processing has been well-established in pain management and pharmacological research (56). However, the specific activation status of microglia in a hyperglycemia peripheral nerve environment and their subsequent pain-mediating actions remain elusive. Hyperglycemia not only triggers microglial activation but also induces dynamic morphological remodeling, as evidenced by enhanced Iba1 immunoreactivity observed in the medial region of the spinal dorsal horn, exhibiting characteristic cellular hypertrophy (57). Furthermore, heightened phosphorylation of the N-methyl-D-aspartate receptor (NMDAR) NR1 subunit (pNR1) on microglia has been linked to the development of mechanical hyperalgesia in diabetic mice (58).

DNP predominantly arising from peripheral nerve damage, is also significantly modulated by the central nervous system’s regulatory dysfunction. This central dysregulation in turn induces abnormalities in peripheral sensory neurons (59). Microglia are instrumental in the etiology of DNP and engage in immune regulation, inflammatory responses, neurotransmitter imbalances, and receptor activation, such as the inhibition of P2X4 and Axl receptors (30, 60). These processes can precipitate abnormal neuronal firing and the phagocytosis of myelin debris, as well as the release of insulin-like growth factors (61, 62). Even in the early stages of diabetes, microglia are activated by elevated blood glucose levels, leading to a notable proliferation within the spinal cord’s gray and white matter. Activated microglia exhibit ultrastructural changes, including an abundance of endoplasmic reticulum and lysosomal fragments and an increase in nuclear-associated actin filaments. Functionally, they demonstrate heightened phagocytic activity, evident by the presence of myelin and axonal debris within their cytoplasm (63). Bullatine A demonstrates consistent analgesic efficacy across preclinical neuropathic pain models, encompassing spinal nerve ligation, cancer-induced pain, and diabetic neuropathic pain (DNP). This pharmacological action is primarily mediated through microglial activation within the superficial dorsal horn laminae, triggering subsequent dynorphin A release. Notably, Bullatine A’s analgesic effects are abolished by κ-opioid receptor antagonism or dynorphin A neutralization (37, 64). Sun et al. (65) have validated that C18-diterpene alkaloids primarily target microglia over neurons and astrocytes to mitigate pain hypersensitivity. Morevermicroglia-derived C-class chemokine XCL1 plays a pathogenic role in the neuroimmune pathogenesis of diabetic neuropathic pain (DNP) (40).

### 2.2 Astrocytes play a crucial role in diabetic neuropathic pain

Astrocyte activation within the spinal cord is a pivotal factor in the development of diabetic neuropathic pain (DNP). Recent findings through single-cell sequencing and spatial transcriptomics have revealed co-localization of C4b with astrocytes and GABAergic neurons in mice subjected to a high-fat diet (66). It has been established that astrocyte activity is markedly diminished in hyperglycemia conditions (67, 68). Ge et al. (47) has demonstrated that dihydromyricetin mitigates the deleterious effects of hyperglycemia on astrocytes and decreases the expression of inflammatory cytokines such as TNF-α and IL-1β by targeting the purinergic receptor P2X7. Astrocyte activation not only elevates inflammatory cytokine levels but also has a direct link to neuropathic pain, as evidenced by the phosphorylation of c-Jun N-terminal kinase (pJNK) in astrocyte (46).

The identity of the cell types in the spinal cord dorsal horn and peripheral nerves that mediate DNP remains a contentious issue across various studies. Liao et al. (69) discovered that the administration of l-α-aminoadipate, an astrocyte-specific inhibitor, significantly reduced mechanical allodynia in type 2 diabetic mice, whereas minocycline, a microglia-specific inhibitor, did not alleviate this symptom. This finding underscores the importance of astrocyte activation in the spinal cord in the pathogenesis of DNP. Activated astrocytes are posited to propagate pain signals by increasing IL-1β levels and NMDA receptor phosphorylation in dorsal horn neurons (70). In contrast to research on spinal glial cells, some researchers propose that alterations in neurotransmitter and receptor expression within the ventrolateral periaqueductal gray (vlPAG) are crucial for endogenous pain modulation (71, 72). Studies have indicated that astrocyte activation in the vlPAG is closely associated with the mechanical withdrawal threshold (MWT) in DNP rats, and treatment with fluorocitrate (FC), a selective astrocyte activation inhibitor, or neurotropin analgesics can ameliorate MWT (41). Additionally, Lan et al. confirmed that vlPAG astrocytes regulate diabetes-associated neuropathic pain and related anxiety-like behaviors (45). The most recent research (42) employs chemogenetic methods to show that the Designer Receptors Exclusively Activated by Designer Drugs (DREADD) can bidirectionally regulate astrocyte activation in the basolateral amygdala (BLA), with activation alleviating and inhibition exacerbating mechanical allodynia in rats.

### 2.3 Schwann cells mediate the mechanisms of diabetic neuropathic pain

Schwann cells, the principal glial cells of the peripheral nervous system, perform critical functions such as providing trophic support and nutrition to neurons, ensheathing them to form myelin, thereby enhancing nerve impulse propagation, fostering axonal regeneration, and engaging in immune modulation (73–75). In the clinical context of DNP, initial structural and functional impairments encompass axonal defects termed axonopathy and pathological changes within Schwann cells, referred to as Schwann cell disease. These cells are pivotal in the pathogenesis of DNP, underscoring their essential role in peripheral nerve support. In 2013, Zenker (76) put forth a notable hypothesis regarding the neurochemical mechanisms underlying DNP, positing that Schwann cells, along with dorsal root ganglion neurons and spinal oligodendrocytes, are integral mediators of neuropathic pain.

Peripheral nerve damage is a fundamental pathological underpinning of dDNP (77). Prolonged hyperglycemia in diabetes engenders oxidative stress and amplifies inflammatory responses, precipitating aberrant peripheral nerve conduction and pain sensation (78). Hyperglycemia impedes Schwann cell secretion of nerve growth factor (NGF) and neurotrophin 3 (NT3), curtailing the neurotrophic support essential for axonal health and impeding regeneration (79). Zheng et al. (80) have validated that hyperglycemia-induced Schwann cell pyroptosis potentially contributes significantly to neuropathy, and modulation of systemic inflammatory factors can avert this pyroptosis, safeguarding neuronal integrity. Recent studies have indicated that bupropion, an antidepressant medication, is associated with a diminished prevalence of neuropathy among diabetics; experimental models in mice have corroborated that bupropion mitigates Schwann cell mortality and upholds myelin integrity (81). Consequently, maintaining the integrity of peripheral nerves is of utmost importance in alleviating and preventing DNP. This involves focusing on reducing abnormal nerve signal conduction, which is crucial for minimizing the development and progression of the condition.

### 2.4 Neurons involved in regulation of diabetic neuropathic pain

The dorsal root ganglion (DRG) serves as the sensory nexus for spinal nerves, harboring pseudo-unipolar neurons that are pivotal for conveying sensory information from the periphery to the central nervous system (82). Pathophysiological alterations within the DRG encompass neuronal hyperactivity, neurotransmitter dysregulation, and escalated pain signal conduction—factors intimately associated with hyperalgesia and chronic pain conditions (83). Research indicates that DRG neurons in diabetic patients are frequently subjected to metabolic stress, rendering them more vulnerable to the detrimental impacts of hyperglycemia (84). Notably, the large, myelinated A-fiber neurons within the DRG, particularly Aβ fibers, are instrumental in pain signal transmission (85, 86). Elevated plasma osmotic pressure in diabetic patients, a consequence of hyperglycemia, can result in diminished peak amplitude of the ascending compound action potential (CAP) in A fibers. This heightened osmotic pressure can precipitate paresthesia and chronic pain by disrupting signal transduction within A fibers. Xu et al. (87) have discovered that toll-like receptor 5 (TLR5) co-localizes with A-fiber neurons in the DRG, and local anesthetic agents can impede TLR5-mediated sodium currents. This inhibition is achieved by activating TLR5 and its ligand, flagellin, thus mitigating diabetic neuropathy. Current research further validates the significant role of Aβ low-threshold mechanoreceptors (Aβ-LTMRs) in mechanical hyperalgesia, with varying degrees of receptor activation and deactivation dictating the facilitation or suppression of mechanical hyperalgesia (88).

The transient receptor potential vanilloid type 1 (TRPV1) is an ion channel belonging to the transient receptor potential (TRP) superfamily of cation channels (89). It is notably expressed in the DRG, where its blockade can effectively reduce the transmission of pain signals. TRPV1 also exerts regulatory roles in the central nervous system, implicating it in a spectrum of diseases including neurodegeneration, obesity, and diabetes (90–92). Notably, it is particularly abundant in the spinal cord’s dorsal horn, where it modulates peripheral pain signal transmission. An abundance of research has documented elevated expression levels of TRPM8 (transient receptor potential melastatin 8) in the spinal cord and DRG of neuropathic pain animal models, positing TRPM8 as a promising therapeutic target (93, 94). Adnan Khan et al. reported a significant upregulation of spinal TRPV1/TRPM8 proteins in a streptozotocin-induced diabetic model. Treatment with Aju-I markedly attenuated the expression of these proteins in the spinal cord of mice. These findings align with previous studies, suggesting that the pharmacological targeting of TRPV1/TRPM8 could mitigate hyperalgesia in models of diabetic neuropathy (95–97).

Research has confirmed that the activation of transient receptor potential vanilloid 1 (TRPV1) on A-fiber neurons plays a critical role in the mechanism of pain amplification (98). Recent investigations have identified that GPR177 is predominantly expressed in large-caliber A-fiber DRG neurons (99). It has been elucidated that GPR177 facilitates the secretion of WNT5a into the cerebrospinal fluid (CSF) by A-fiber DRG neurons, a process imperative for sustaining DNP. *In vitro* experimentation has corroborated that the extracellular application of WNT5a elicits rapid currents in both heterologous cells that express TRPV1 and in nociceptive DRG neurons (99). Administration of Aju-I has been shown to significantly diminish the expression levels of TRPV1 and TRPM8. Furthermore, Aju-I augments antioxidant capacity and curbs the secretion of inflammatory cytokines. Collectively, this research delineates that Aju-I mitigates pain behaviors in the streptozotocin (STZ)-induced diabetic neuropathy model by modulating the Nrf2/Keap-1/HO-1 signaling pathway and the activity of TRPV1/TRPM8 nociceptors (100).

## 3 The role and regulation of ion channels in diabetes-induced neuropathic pain

### 3.1 Sodium channels in diabetic neuropathic pain

Sodium channels are a primary therapeutic target for the development of novel analgesics aimed at neuropathic pain management (101). Nerve injury can modulate the expression of sodium channels, which may lead to heightened peripheral nerve excitability and the generation of ectopic discharges at the nerve, dorsal root ganglion, or site of injury (102). These channels consist of an α subunit that forms the pore, associated with one or two β subunits of a regulatory nature. The α subunits are encoded by nine genes, namely Nav1.1 through Nav1.9 (103, 104). Sodium channel inhibitors, such as amitriptyline and mexiletine, among other anticonvulsant medications, have clinically demonstrated efficacy in alleviating neuropathic pain by promoting the stabilization of the inactivated state of sodium channels (105).

Differential alterations in sodium channels within small nociceptive C-fiber DRG neurons are implicated in the pathogenesis of diabetic neuropathy (106, 107). Research has established that early-stage diabetic conditions are characterized by modifications in nanochannels of large DRG neurons and myelinated A-fibers (108). In diabetic mice, the expression of Na(v)1.2, Na(v)1.3, Na(v)1.7, and Na(v)1.9 in DRG neurons was increased, accompanied by a significant rise in sodium currents and the mean peak current density of the slow ramp current. Conversely, at the sodium channel-rich nodes of Ranvier, the signal intensity of Na(v)1.6 was reduced, and the quantity of Na(v)1.8 was significantly decreased. Therefore, the differential expression of sodium channels in large DRG neurons and A-fibers may be a key factor mediating diabetic neuropathic changes (108). Additionally, alterations in sodium channel proteins in medium or small DRG neurons can also contribute to abnormal sensory conduction in the early stages of diabetes (109). Another study (110) found that in diabetic rats, the expression of tetrodotoxin-sensitive (TTX-S) sodium channels Na(v)1.3 and Na(v)1.7 was significantly increased, while the expression of Na(v)1.6 (TTX-S) and Na(v)1.8 (tetrodotoxin-resistant, TTX-R) was decreased. Phosphorylation of sodium channels can enhance sodium current levels (111). Consequently, the study further revealed that serine/threonine phosphorylation levels of Na(v)1.6 and Na(v)1.8 were elevated by more than 60%. Simultaneously, the anti-phosphotyrosine immunoreactivity levels of Na(v)1.6 and Na(v)1.7 increased by approximately 80%. These findings suggest that not only differential expression of sodium channels but also their differential phosphorylation at serine/threonine and tyrosine residues plays a significant role in the etiology of diabetic painful neuropathy. Phosphorylation of Na(v)1.8 is associated with increased excitability and pain sensitization in DRG neurons of diabetic rats. Rapamycin can inhibit mTOR and Na(v)1.8 phosphorylation, thereby reducing current density and voltage thresholds and alleviating hyperalgesia (112). A novel pyridine derivative has shifted the traditional frequency-dependent inhibition of currents to a voltage-dependent inhibition of TTX-R currents, providing relief from tactile allodynia (113) (**Table 2**).

**Table 2:** Mechanisms of ion channel regulation in diabetic neuropathic pain.

Ion Channel	Year	Intervention	Model	Mechanism	References
Sodium Channel	2019	/	STZ Model	Upregulation of NaV1.7, increased total sodium current, TTXs sodium current, and TTXr sodium current density	(114)
Sodium Channel	2015	Curcumin	HFD Model	Inhibition of current density and TTX-R sodium currents in small-sized DRG neurons	(115)
Sodium Channel	2011	/	db/db/db/+	Increase in Nav1.6 protein and mRNA expression; increase in Nav1.6 positive cells proportion	(116)
Sodium Channel	2004	/	STZ Model	Decrease in the expression of Na(v)1.6 (TTX-S) and Na(v)1.8 (TTX-R), increase in tyrosine phosphorylation of Na(v)1.6 and Na(v)1.7, significant increase in both TTX-S and TTX-R sodium currents in small DRG neurons isolated from diabetic rats	(110)
Sodium Channel	2015	AAV-shRNA-Nav1.3	STZ Model	Downregulation of expression of Nav1.3, reduced neuronal excitability of dorsal horn neurons	(117)
Sodium Channel	2012	/	STZ Model	Upregulation of expression of Nav1.7 and Nav1.8; enhancement of transient and persistent sodium currents, increase in neuronal excitability	(106)
Potassium Channel	2024	Branched-Chain Amino Acids	HFD/STZ/db/db Model	Activation of expression of L-type amino acid transporter 1 (LAT1), decreased localization of Kv1.2 on the cell membrane, inhibition of Kv1.2 channels, increased neuronal excitability	(118)
Potassium Channel	2015	α-dendrotoxin (α-DTX)	STZ Model	Reduction in conduction failure of C-fibers; decrease in expression of Kv1.2 and Kv1.6	(119)
Potassium Channel	2018	/	STZ Model	Significant reduction in mRNA and protein levels of KCNQ/3/5 channels, decreased IM density, increased excitability of DRG neurons	(120)
Potassium Channel	2010	/	STZ Model	Marked reduction in densities of total Kv, A-type (IA), and sustained delayed (IK) currents in medium- and large-, but not in small-diameter DRG neurons	(121)
Calcium Channel	2022	/	STZ/db/db Model	Activation of GPR77 receptor mediates WNT5a release followed by activation of TRPV1 channel, induction of rapid currents	(99)
Calcium Channel	2022	/	STZ Model	Activation of PKCϵ/P38 MAPK/NF-κB signaling pathway increases TRPV1 expression	(122)
Calcium Channel	2023	Selenium, Curcumin	STZ Model	Reduction in mean densities of TRPM7 currents, increase in calcium concentration	(123)
Calcium Channel	2023	Alpha-lipoic acid	STZ Model	Reduction in TRPV1 current density and intracellular free Ca^2+^ concentration	(124)
Calcium Channel	2023	Alpha-lipoic acid	STZ Model	Inhibition of TRPV4 channel, reduction in intracellular free Ca^2+^ concentration	(125)
Calcium Channel	2017	Melatonin, Selenium	STZ Model	Reduction in current densities of TRPM2 and TRPV1 channels, decreased intracellular free Ca^2+ concentration	(126)

### 3.2 Calcium channels in diabetic neuropathic pain

Under hyperglycemia conditions, the capsaicin-induced calcium (Ca2+) influx in DRG neurons is diminished, potentially a significant contributor to the accumulation of reactive oxygen species (ROS) within these cells (127). The precise role of TRPV1 in mediating neural injury is still under investigation. Previous research has indicated that capsaicin can trigger a reduction in MitoTracker Red fluorescence, an elevation in cytosolic cytochrome c, and the activation of caspase 3, all of which are indicative of heightened oxidative stress (128). Utilizing TRPV1 antagonists, the expression of mu-calpain and the heightened calpain activity observed in DRG neurons of diabetic rats were effectively mitigated. It is thought that the activation of TRPV1 receptor on neuropathy relatively early leads to the preference of large DRG neurons over neuronal stress at the early stages of neuropathy (128). Cao et al. have demonstrated that diabetic rats exhibit high-voltage-activated calcium channel (HVA) currents in larger neurons, as well as T-type currents in medium to large neurons and small DRG neurons (129). There was a notable increase in the expression of Cav3.2 mRNA and the proportion of its corresponding elements. Antagonizing the M4 receptor can significantly attenuate the calcium inflow current, offering pain relief.

### 3.3 Potassium channels in diabetic neuropathic pain

**Figure 3:**
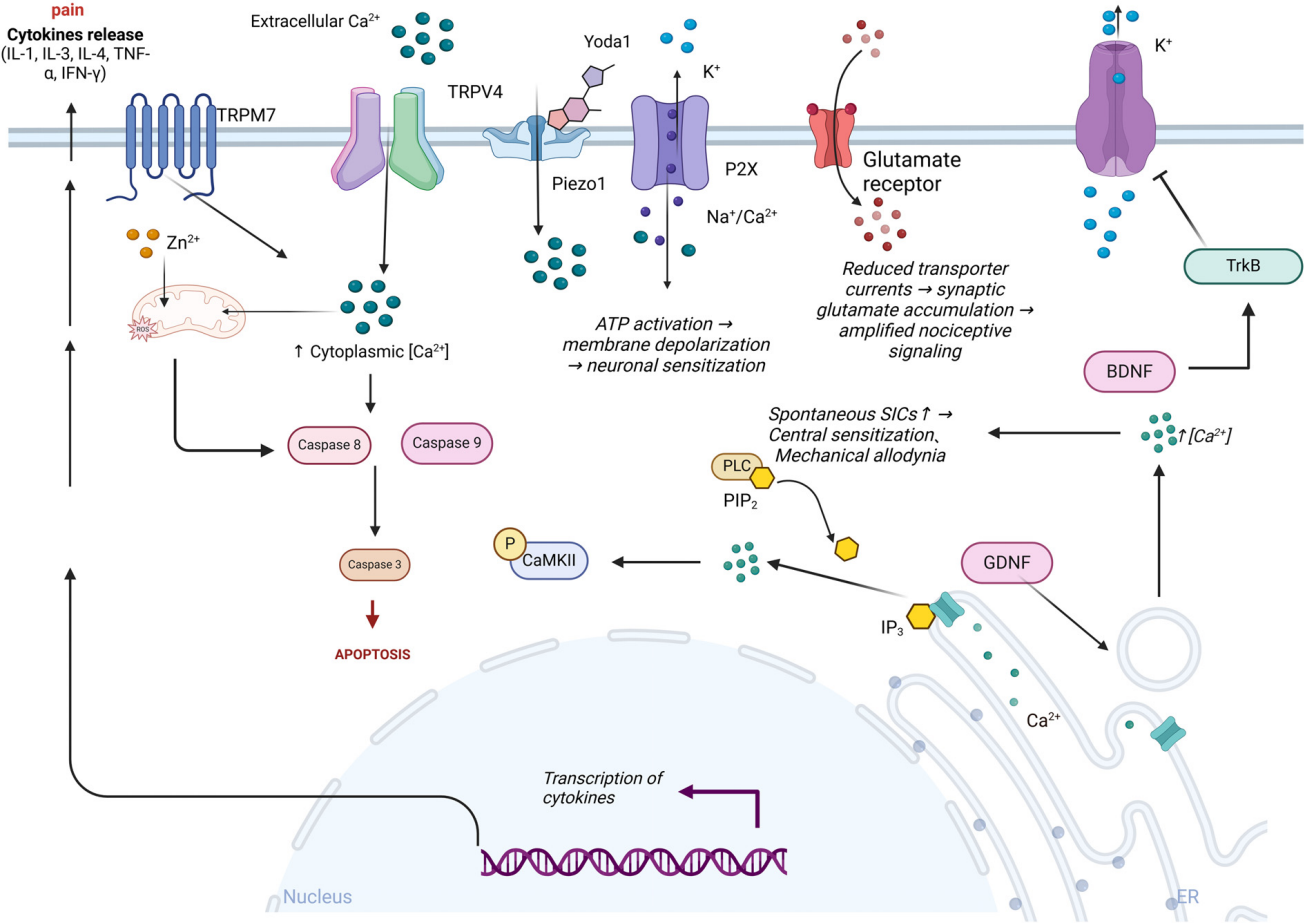
Ion channels’ role in the pathogenesis of diabetic neuropathic pain. Extracellular calcium ions (Ca²⁺) activate TRPM7 and TRPV4 channels, leading to an increase in intracellular calcium levels, which in turn activates caspase 8 and caspase 9, promoting cell apoptosis. Intracellular reactive oxygen species (ROS) regulate the opening of ion channels, exacerbating oxidative stress and promoting the transcription and release of cytokines, thereby intensifying pain. Following Yoda1 activation of the mechanosensitive Piezo1 channel, ATP release exacerbates K⁺ efflux and P2X receptor-mediated Na⁺/Ca²⁺ influx, leading to inhibition of glutamate transporter function and amplification of nociceptive signaling. Concurrently, the PLC-IP3 pathway triggers endoplasmic reticulum Ca²⁺ release via hydrolysis of PIP₂, enhancing spontaneous slow inward currents (SICs) and inducing central sensitization and mechanical allodynia. BDNF alleviates hyperalgesia by activating the TrkB receptor and inhibiting potassium ion efflux.

Voltage-dependent potassium channels (KCNQ) are extensively expressed in dorsal root ganglia and play a pivotal role in modulating neuronal excitability (**Figure 3**). Studies have established that the inhibition of the KCNQ pathway can significantly curtail the influx of potassium ions, attenuate signal transmission, and provide substantial relief from peripheral nerve damage and the activation of inflammatory mediators. KCNQ channels are instrumental in generating a slow, non-inactivating potassium current, commonly referred to as the M current (IM). They become active within the subthreshold range of membrane potential and influence various facets of neuronal excitability (130). In diabetic rat models, there is a notable reduction in the expression levels of KCNQ2/3/5 channel proteins in DRG neurons, along with a decrease in IM density. Concurrently, recordings under current clamp conditions have documented depolarization of the resting membrane potential (RMP), a reduction in action potential (AP) threshold, and an increase in AP frequency, indicative of heightened neuronal excitability. This increase in excitability is thought to augment mechanical allodynia and thermal hyperalgesia associated with diabetic neuropathic pain (120). However, not all neurons exhibit altered potassium channel expression; it has been confirmed that Kv7.5 predominantly diminishes immunoreactivity in small to medium-sized nerve fibers of less than 40μm, while Kv7.2 mainly reduces immunoreactivity in neurons less than 30μm. The downregulation of potassium ion channel proteins is believed to contribute to the over-excitability mechanism of neurons (131). Leish Juchri et al. has identified the activation of the Kv7 pathway as a significant biological target for the retigabine (ezogabine)-mediated reduction of mechanical hypersensitivity in diabetic peripheral neuropathy (DPN) mouse models, and when the Kv7/M channel is obstructed, the analgesic effect of ezogabine is significantly diminished (132).

Following nerve injury, the current densities of various types of potassium currents, including sustained current (I(K)), transient current (I(A)), and I(D), were significantly reduced in medium and large DRG neurons. Additionally, the mRNA levels of I(A) subunits, including Kv1.1, Kv1.2, Kv1.3, and Kv1.4, were decreased by approximately 35-80% (133, 134). Potassium channels are positioned as promising therapeutic targets for the treatment of neuropathic pain. Voltage-gated potassium channel α-subunits are critical targets mediating central sensitization and pain regulation mechanisms (135). Studies have shown that the miR-17-92 cluster is widely expressed in DRG neurons following injury. The miR-17-92 cluster reduces outward potassium currents, particularly A-type currents, by targeting and suppressing the expression of α-subunits, ultimately leading to altered neuronal excitability. Furthermore, the administration of potassium channel modulators (flupirtine and NS5806) effectively alleviated mechanical hyperalgesia induced by miR-17-92 overexpression (136). In diabetic rats, the total voltage-gated potassium channel (Kv) current density, A-type current (I(A)), and delayed rectifier potassium current (I(K)) peak density were significantly reduced in medium- and large-diameter DRG neurons, while no significant differences were observed in small-diameter neurons (121). Concurrently, the mRNA levels of I(A)-related subunits (including Kv1.4, Kv3.4, Kv4.2, and Kv4.3) in DRGs of diabetic rats were approximately halved compared to the control group (121). Therefore, diabetes may primarily induce peripheral pain abnormalities by impairing Kv channel function in medium- and large-diameter DRG neurons.

## 4 Ion channel-mediated pain regulatory mechanisms in neurons and glial cells

### 4.1 Astrocytes and ion channel regulation

Alterations in astrocytic ion channels within the spinal cord play a pivotal role in mediating neuropathic pain. Ligand-gated cationic channels such as P2X receptors permit transmembrane flux of Na⁺, Ca²⁺, and K⁺. As non-selective cation channels, P2X receptors mediate ATP-dependent Na⁺/Ca²⁺ influx and K⁺ efflux, inducing membrane depolarization and enhancing neuronal excitability, thereby contributing to peripheral and central sensitization in chronic pain (137). The normal function of glutamate transporters on astrocytes relies on Na⁺ influx, with glutamate transporter currents reflecting electrogenic glutamate uptake. Reduced glutamate transporter currents indicate synaptic glutamate accumulation, amplifying pain signal transmission (138). Lundborg et al. (139) demonstrated that glial cell line-derived neurotrophic factor (GDNF) induces intracellular Ca²⁺ transients in astrocytes via receptor binding by using the Ca²⁺-sensitive fluorescent probe fura-2 AM, and Ca²⁺ transients may disrupt astrocytic function, exacerbating neuroinflammation and pain. The inwardly rectifying potassium channel protein 4.1 (Kir4.1) is specifically expressed in astrocytes, conditional knockout of spinal astrocytic Kir4.1 induces hyperalgesia, while its overexpression alleviates pain (140). Kir4.1 downregulation not only enhances neuronal excitability but also promotes glutamate release from both astrocytes and neurons, further amplifying nociceptive signaling (141). GABA release modulates neuronal excitability, influencing sensory processing and pain transmission. Studies reveal that glutamate binding to astrocytic receptors elevates intracellular Ca²⁺, triggering GABA release—a process blocked by Ca²⁺ chelation in astrocytes (142). Orai1, a core component of calcium release-activated calcium (CRAC) channels, mediates Ca²⁺ entry into astrocytes (143). Birla et al. (144) found that Toll-like receptor 4 (TLR4) upregulates astrocytic Orai1 expression, enhancing Ca²⁺ influx. Notably, Orai1 knockout astrocytes exhibit >50% reduction in inflammatory cytokines, positioning Orai1 as a therapeutic target for chronic pain. In neuropathic pain models, astrocytic KATP channel subunits (SUR1, SUR2, Kir6.1) show marked protein downregulation. Pharmacological activation of KATP channels with cromakalim dose-dependently prevents or suppresses hyperalgesia and allodynia in model rats, concurrently inhibiting NMDA receptor hyperactivation, Ca²⁺-dependent signaling pathways, and modulating astrocytic connexin 43 expression to alleviate neuropathic pain (145). Furthermore, astrocyte-neuron crosstalk manifests as Ca²⁺-elevation-induced slow inward currents (SICs) in lamina II neurons, with increased spontaneous SICs correlating with hyperalgesia and mechanical allodynia progression (146). Thus, astrocytic ion channels directly or indirectly regulate pain pathways, and their precise roles in DNP require further investigation.

### 4.2 Microglia and ion channel regulation

Intrathecal administration of the sodium channel activator BmK I in rats upregulated P2X7R expression on microglia in the spinal dorsal horn, accompanied by elevated pro-inflammatory cytokine levels, and pharmacological blockade of P2X7R using brilliant blue G (BBG, a P2X7R antagonist) significantly alleviated spontaneous pain behaviors and thermal hyperalgesia (147). Although this study did not directly assess sodium flux changes induced by BmK I, prior reports indicate that BBG exhibits sodium channel inhibitory activity. N-type voltage-dependent calcium channels (VDCCs), particularly Cav2.2, are critical regulators in the initiation and maintenance of neuropathic pain (148). Japanese researchers (149) observed that conditional Cav2.2 knockdown mice showed markedly reduced tactile allodynia following spinal nerve ligation (SNL) injury, though thermal hyperalgesia remained unaffected. Additionally, Cav2.2 knockdown reduced microglial proliferation in the spinal cord. Upregulation of P2X4 receptors in spinal microglia is implicated in neuropathic pain development. Taspine attenuates neuropathic pain by inhibiting P2X4 receptor activity, impairing microglial function, and reversing the enhancement of ATP-induced calcium signaling by ivermectin (a P2X4 receptor positive allosteric modulator). Mechanistically, Taspine suppresses microglial inflammatory responses via P2X4 receptor inhibition. The Ca²⁺-activated potassium channel KCa3.1 regulates microglial physiological functions by facilitating K⁺ efflux, which induces membrane hyperpolarization, paradoxically promoting Ca²⁺ influx and sustaining elevated intracellular Ca²⁺ concentrations (150). Administration of the KCa3.1 channel inhibitor senicapoc significantly reversed mechanical hyperalgesia in rats with peripheral nerve injury while suppressing microglial activation and inflammatory factor release (151). BK channels, another class of Ca²⁺-activated potassium channels, are activated by increased intracellular Ca²⁺ in microglia following nerve injury, promoting K⁺ efflux to modulate cellular excitability and neurotransmitter release. In neurons, BK channels inhibit action potential generation via membrane hyperpolarization, thereby reducing neuronal excitability. However, specific knockdown of the Ca²⁺-activated potassium channel β3 auxiliary subunit (KCNMB3) suppresses BK channel activation in microglia, attenuating microglial activation and pain-related molecule release, ultimately alleviating hyperalgesia (152). Thus, BK channel activation in microglia enhances pain signaling not through hyperpolarization-mediated excitability suppression but by promoting microglial activation and pain-enhancing molecule release. Ketamine, a clinically used analgesic for both acute and chronic pain, exhibits distinct mechanisms, its acute analgesic effects primarily arise from NMDA receptor inhibition in neurons (153), whereas its chronic pain-modulating mechanisms remain controversial. Lin et al (154). demonstrated that S-ketamine suppresses BK channel currents in microglia, reducing pathological microglial activation, inflammatory cytokine release, P2X4 receptor expression, and BDNF synthesis, thereby mitigating chronic neuropathic pain.

### 4.3 Schwann cell and ion channel regulation

Piezo1 and Piezo2 are mechanosensitive ion channels (MSCs) widely expressed throughout the body. Recent studies reveal that Piezo1 is highly expressed not only in neurons but also in Schwann cells. Under stimulation by the Piezo1 agonist Yoda1, Schwann cells exhibit increased intracellular calcium concentration ([Ca²⁺]i), suggesting a critical role for Schwann cell Piezo1 in mechanosensation and pain transduction (155). In a separate investigation, researchers explored Piezo2 regulation in Schwann cells, demonstrating its predominant expression in differentiated Schwann cells. Piezo2 mediates mechanical stimulus detection (e.g., cellular swelling) to trigger Ca²⁺ influx, thereby regulating cell volume and neurotrophic factor release, and Piezo2 deficiency paradoxically enhances TRPV4 channel activity, exacerbating calcium overload (156). Human tonsil-derived mesenchymal stem cells can differentiate into Schwann-like cells (NRPCs), which may alleviate hyperalgesia by secreting neurotrophic factors such as BDNF and glial cell line-derived neurotrophic factor (GDNF). These factors not only promote neural regeneration but also modulate TRPV1 expression in DRG neurons (51). Schwann cells express diverse potassium channels, including A-type (KA), delayed rectifier (KD), and inwardly rectifying (KIR) subtypes. Delayed rectifier potassium currents are generated by heteromeric complexes of Kv channel α-subunits. Broad-spectrum potassium channel blockers (e.g., quinine, 4-aminopyridine, quinidine) effectively inhibit Schwann cell proliferation, whereas selective Kv1.1, Kv1.2, Kv1.3, and Kv1.6 channel toxins like dendrotoxin I (DTX) show no such inhibitory effects (157). Schwann cells are classified into myelinating and non-myelinating subtypes, with the latter being crucial for maintaining neural functionality. Notably, Kir4.1 is specifically expressed and functionally active in non-myelinating Schwann cells, where it regulates potassium uptake to modulate membrane potential and neural signaling. This mechanism may contribute to pain perception and related pathologies, offering novel directions for therapeutic exploration (158).

### 4.4 Neuron and ion channel regulation

The Kv4 channel complex consists of pore-forming subunits (Kv4.1-Kv4.3) and two regulatory subunits (KChIP1-4 and DPP6/DPP10). Previous studies confirmed that Kv4.3 knockout selectively induces mechanical hypersensitivity (159). Guo et al. (160) demonstrated that knockdown of any component of the Kv4 complex (Kv4.3, KChIP1, KChIP2, or DPP10) reduces the expression of other subunits, induces mechanical hypersensitivity, and enhances excitability in IB4⁺ nociceptors. Regulatory subunits of Kv4 channels represent promising therapeutic targets for precision treatment of neuropathic pain. In contrast to other ion channels, Kir7.1 is predominantly localized to neuronal membranes rather than glial cells in rats. Co-immunoprecipitation revealed that spinal Kir7.1 channels undergo SUMO-1 modification but not SUMO-2/3 conjugation, with SUMOylation upregulated following spared nerve injury (SNI). Furthermore, pharmacological inhibition of SUMOylation using ginkgolic acid (GA, an E1 inhibitor) or 2-D08 (a UBC9 inhibitor) increases surface expression of Kir7.1 in the spinal cord (161), highlighting Kir channels as potential therapeutic targets for pain management (162). Nav1.8, a voltage-gated sodium channel selectively expressed in peripheral nociceptors, is enriched in DRG neurons (163). In a Phase III clinical trial involving 2,447 participants, suzetrigine significantly alleviated moderate-to-severe acute pain by selectively inhibiting Nav1.8-mediated pain signaling in the peripheral nervous system (164). Similarly, Nav1.7, another voltage-gated sodium channel essential for initiating C-fiber action potentials (APs), confers pain insensitivity in Nav1.7 knockout (KO) mice or upon pharmacological inhibition (165). Huang et al (166). reported that the Nav1.7 I234T mutation depolarizes the resting membrane potential (RMP) in a subset of DRG neurons, rendering them electrically silent and incapable of generating APs, thereby causing congenital pain insensitivity. Conversely, milder RMP depolarization in other neurons increases neuronal excitability, driving pain phenotypes. Intracellular calcium concentration ([Ca²⁺]i) in DRG neurons is critically associated with neuropathic pain. Lamotrigine elevates [Ca²⁺]i by activating the PLC-IP3R/RyR signaling pathway and stimulating calmodulin-dependent kinase II (CaMKII). This [Ca²⁺]i increase primarily originates from intracellular Ca²⁺ release rather than extracellular Ca²⁺ influx (167).

## 5 Herbal extracts in diabetic neuropathic pain management

The etiology of DNP is intricate, and there exists a dearth of efficacious therapeutic agents and modalities (168). DNP significantly impairs patients’ quality of life, with studies indicating that persistent chronic pain adversely affects mental well-being, further impacting their daily routines (169, 170). Despite extensive research highlighting that DNP involves a complex interplay of factors in aberrant signal transduction (171, 172), the pathophysiological intricacies present a formidable challenge in the development of potent medications. Presently, the US FDA has approved three medications for DNP treatment: pregabalin, duloxetine, and the 8% capsaicin patch (173–175). Moreover, amitriptyline, and select opioid analgesics are also utilized clinically to alleviate DNP symptoms (176–178). In severe cases, potent opioids such as fentanyl may be prescribed (179), necessitating a delicate balance between effective pain management and the risks of addiction to prevent patients from succumbing to substance dependence (180).

Additionally, herbal extracts and compound formulations derived from traditional Chinese medicine offer novel avenues for the alleviation of DNP, contributing fresh perspectives for the development of DNP therapeutics and the refinement of treatment protocols (181, 182) (**Table 3**). Jinmaitong, a traditional Chinese medicinal prescription, Zhang and his colleagues has been demonstrated that Jinmaitong, a traditional Chinese medicinal prescription, exhibits efficacy in mitigating microglial activation induced by hyperglycemia curb the activation of inflammatory factors and chemokines, and bolster the expression of anti-inflammatory mediators within the body (183). Quercetin has been shown to suppress the expression of the synaptic protein PSD-95 and the synaptic plasticity of neurons in the dorsal horn, thereby diminishing the propagation of aberrant signals and reducing hyperalgesia (184). Curcumin, with its antioxidant and anti-inflammatory properties, has also proven effective in curbing the release of inflammatory mediators. Korean researchers (46) have confirmed in DNP models that curcumin can alleviate neuropathic pain by suppressing the heightened expression of PJN on astrocytes and neurons. Furthermore, other botanical extracts, such as Emodin, Nerunjil, and Emblica, have been identified to significantly contribute to the inhibition of inflammatory factor release, oxidative stress, and the mitigation of DNP (199, 201).

**Table 3:** Mechanisms of herbal extracts in alleviating diabetic neuropathic pain.

Drug	Year	Dose and Administration	Cell	Target/Pathway	Mechanism/Pathway	References
Jingmaitong	2024	11.6 g/kg, 23.2 g/kg和46.4 g/kg, p.o.	Microglia	JAK2/STAT3	suppression of pro-inflammatory cytokines (TNF-α, IL-1β, IL-6, IL-12, and iNOS), anti-inflammatory cytokines (IL-10, Arg-1, and TGF-β), and chemokines (CCL3, CCL5, and CXCL12)	(183)
Quercetin	2020	50 mg/kg, 100 mg/kg, p.o.	Dorsal horn neurons	TOR/p70S6K	Reduction of synaptic plasticity	(184)
Curcumin	2021	10 mg/kg, 50 mg/kg, p.o.	Astrocytes\neurons	/	Inhibition of JNK activation	(46)
Dihydromyricetin	2019	30mg/kg, i.p.	Astrocytes	P2X7	Inhibition of P2X7 receptor expression, suppression of inflammatory factor release	(185)
Insulin+resveratrol/Insulin+curcumin	2007	20 mg/kg/60 mg/kg, p.o.	/	/	Inhibition of TNF-α and nitric oxide NO	(186)
Resveratrol	2024	25 mg/kg, i.p.	Dorsal root ganglion (DRG)	TRPV4	decrease in [Ca2+]I, downregulation of TRPV4 current densities, ROS, apoptosis, etc.	(124)
Selenium and Curcumin	2023	Se: 1.5 mg/kg + Curcumin: 25 mg/kg, i.p.	Dorsal root ganglion	TRPM7	decrease in [Ca2+]I, upregulation of TRPM7 current density, increased mitochondrial calcium and zinc ion concentrations mediating mitochondrial ROS (mitROS)	(123)
Nanoparticle-Encapsulated Curcumin	2018	16 mg/kg, i.v.	Satellite glial cells	P2Y12	reduction of IL-1β, Cx43, p-Akt levels	(187)
Curcumin	2015	100 mg/kg, i.p.	Dorsal root ganglion	TTX-resistant (TTX-R) sodium channel	Inhibition of TTX-R INa increase in DRG neurons	(115)
Fisetin	2015	10 mg/kg, p.o.	Dorsal root ganglion	GABA	Alleviates oxidative stress (lipid peroxidation, reactive oxygen species, and catalase activity)	(188)
Cannabidiol	2019	3 mg/kg, i.p.	/	5-HT1A	elevates serotonin levels	(189)
Camphor	2023	50 mg/kg, i.p.	Dorsal root ganglion	TRPA1	reduced the action potential (AP) firing frequency	(190)
Secoisolariciresinol diglycoside	2015	10 mg/kg, p.o.	/	/	Reduces lipid peroxidation, catalase activity, and GSH content	(191)
Emblica officinalis	2011	250 mg/kg、500 mg/kg and 1000 mg/kg, p.o.	/	/	Oxidative stress, nitrite levels, and cytokines (TNF-α, IL-1β, and TGF-β1)	(192)
Naringin	2012	20 mg/kg、40 mg/kg and 80 mg/kg, p.o.	/	/	Neural cell apoptosis, enhanced lipid peroxide, nitrite, and TNF-α level	(193)
Coriandrum sativum	2020	100 mg/kg、200 mg/kg and 400 mg/kg, p.o.	Schwann cells	/	Increases antioxidant enzyme levels such as SOD, GSH, inhibits lipid peroxidation	(194)
Kaempferol	2017	5 mg/kg、10mg/kg	/	/	Reduces oxidative and nitrosative stress, also reduces AGEs	(195)
Olea europaea L	2011	100-600 mg/kg, i.p.	/	/	Inhibits caspase 3 activation, reduces Bax/Bcl-2 ratio	(196)
Thymus caramanicus Jalas	2014	50 mg/kg、100 mg/kg、150 mg/kg和200 mg/kg, p.o.	/	/	Inhibits caspase 3 activation, reduces cytochrome c release and Bax/Bcl-2 ratio	(197)
Curcumin	2014	200 mg/kg, p.o.	/	/	Reduces NADPH oxidase subunits p47(phox) and gp91(phox) expression levels, hydrogen peroxide (H2O2) and malondialdehyde (MDA) levels, and increases superoxide dismutase (SOD) activity (P<0.05)	(198)
Nerunjil	2013	100 mg/kg、300 mg/kg	/	/	Reduces oxidative stress levels	(199)
Mangiferin	2024	15 mg/kg、30 mg/kg, p.o.	/	/	Reducing TNF-α, TGF-β1, IL-1β, and IL-6, inhibits oxidative stress	(200)

i.p., intraperitoneal injection; i.v., intravenous injection; p.o., per os.

## 6 Gender differences in diabetic neuropathic pain

Neuropathic pain is associated with various risk factors, including but not limited to age, geographic location, smoking, alcohol consumption, gender, and disease duration (202–204). Among these, gender is an important factor that should not be overlooked in personalized clinical pharmacotherapy. Cardinez et al (205). investigated long-term diabetic patients in Canada and found that over one-third of these patients experienced neuropathic pain, with 42% being female patients, compared to 27% of male patients. Notably, the association of female gender as a risk factor for diabetic neuropathic pain (DNP) remained robust regardless of whether patients had concomitant neuropathy. Alon Abraham reported that women are more sensitive to pain intensity than men, with a 28.3% higher frequency of pain occurrence in females. Additionally, women reported higher pain intensity, with a greater prevalence of severe pain compared to men (206). A genome-wide association study (207) conducted in the UK confirmed that diabetic neuropathic pain is a heritable trait and identified gender-related genetic loci, specifically Chr1p35.1 (ZSCAN20-TLR12P) in males and Chr8p23.1 (HMGB1P46) in females. This represents the first genetic evidence of gender differences in DNP (208), providing a foundation for gender-based pharmacological research and therapeutic strategies for DNP.

Despite the discovery of gender differences in diabetic neuropathic pain (DNP) from clinical and genetic perspectives, the underlying biological mechanisms remain unclear. One hypothesis suggests that the interaction between the immune and nervous systems may be one of the possible mechanisms leading to chronic pain. Adaptive immunity could be a primary factor mediating these gender differences. Previous studies have confirmed that in DNP patients, there is an increased proportion of CD4^+^ central memory T cells, along with a rise in the absolute numbers of monocytes and natural killer cells, which indicate an inflammatory response (209). Although researchers have not differentiated the relative differences in T cells by gender, this suggests alterations in the adaptive immune response related to DNP. Further research is needed to clarify the changes in immune cell composition and clinical features in DNP patients. Sorge et al (210). revealed that mechanical hypersensitivity in female mice primarily relies on adaptive immune cells, whereas male mice depend on glial cells for pain perception. However, this does not imply that glial cells are uninvolved in the pain processes in female mice; such a phenomenon typically occurs when female mice lack adaptive immune cells, possibly due to the significantly higher presence of peripheral resident T cells in female mice compared to their male counterparts (211). Kuhn et al (212). similarly found that when colony-stimulating factor 1 (CSF1) was injected intrathecally into mice, the activation of microglia in male mice was enhanced, while the activation of microglia was less pronounced in female mice who showed a significant increase in regulatory T cells (Tregs). Subsequent research confirmed that Tregs, as inflammation suppressors, alleviate pain hypersensitivity in mice by inhibiting microglial activation. Interestingly, when CD4+ T cells are absent (excluding CD8+ T cells), female mice require 2 to 3 times the analgesic dose compared to male mice. Additionally, the activation of astrocytes plays a crucial role in neuropathic pain. Recent studies have shown that IL-16 expression levels are elevated in a spinal nerve ligation model involving female mice. Knocking down IL-16 or administering FGF22-IN-1 (a CD4 inhibitor) alleviated pain hypersensitivity in these mice. The interaction of IL-16 with CD4 on CD3+ T cells mediates astrocyte activation, representing an important mechanism of neuropathic pain (213). The gender differences in the perception of neuropathic pain involve complex interactions between the immune and nervous systems (214). The adaptive immune system plays a key role in these sex differences, and the roles of glial cells also vary between genders. More research is needed to explore these mechanisms in depth, to enhance our understanding and treatment of DNP.

Gender differences in neuropathic pain may arise from ion channel mechanisms. Orai1, a core component of calcium release-activated calcium (CRAC) channels, serves as a critical regulator of calcium influx (215). Recent studies demonstrate that conditional knockout of microglial Orai1 in male mice suppresses microglial proliferation, reduces spinal levels of cytokines including TNFα, IL-6, IL-1β, and BDNF, and inhibits excitatory synaptic transmission in dorsal horn neurons, whereas female mice exhibit no significant alterations in these phenotypes (216). In males, microglia-derived BDNF drives neuronal hyperexcitability and mechanical allodynia through KCC2 downregulation, whereas chloride imbalance in females predominantly involves adaptive immune cell pathways rather than microglial mechanisms (210). Yang et al. (217) revealed that TRPA1 and TRPV1 activation induces pain hypersensitivity via TRPM8-dependent release of inflammatory neuropeptides (CGRP and substance P), with notable sexual dimorphism, CGRP exclusively mediates aberrant pain in female mice, and CGRP receptor antagonists specifically reverse cold hypersensitivity in neuroinflammatory female models. Conversely, male mice cold allodynia operates through TLR4 signaling, with TLR4 inhibition providing male-specific analgesia. Furthermore, the “Sex Hormone-ThermoTRP Axis” proposed by Cabañero’s team delineates how sex hormones differentially regulate ion channel functionality and immune signaling networks to mediate pain dimorphism, establishing a molecular framework for gender-specific analgesic strategies (218). Sexual dimorphism in pain represents a frequently overlooked biological determinant in mechanistic research and therapeutic development. Elucidating the dynamic interactions among hormones, ion channels, and immune cell characteristics will advance our understanding of sex-specific pain pathophysiology and facilitate the development of precision-targeted therapeutic interventions.

## 7 Conclusion

Diabetes, characterized by chronically elevated blood sugar levels, is emerging as a significant global health concern due to its rising prevalence (219). Over recent decades, the advancement of public health education and the dissemination of fundamental diabetes knowledge have led to an increased societal understanding of the condition. Adherence to a proper lifestyle and consistent medication use can effectively maintain blood sugar levels within a normal and stable range (220),and this has fortified the practical foundation for patient-centered diabetes management strategies that emphasize prevention and control (221, 222). Nonetheless, despite well-managed blood sugar levels, diabetic patients remain susceptible to complications that can severely diminish their quality of life. In particular, DNP may develop early in the course of the disease (223, 224) and persist even when blood sugar levels are stable, manifesting as symptoms such as limb paresthesia, spontaneous pain, and hyperalgesia (225). This indicates that the pathogenesis of DNP is not solely attributable to hyperglycemia and underscores the need for a deeper investigation into its underlying mechanisms to address clinical challenges (226). In clinical practice, some patients lack a comprehensive understanding of DNP and are even unaware of its connection to diabetes (227). Consequently, when faced with painful clinical manifestations, they may exhibit a dismissive attitude, which can dampen their engagement in DNP treatment.

Recent studies on the mechanistic connections between existing analgesic drugs and ion channel-targeted therapeutic strategies have demonstrated that the analgesic effects of conventional medications extend beyond classical neurotransmitter modulation, involving intricate interactions with ion channels. Pregabalin, a first-line treatment for diabetic neuropathic pain (DNP), exerts its primary action by binding to the α2δ subunit of voltage-gated calcium channels (VGCCs), thereby reducing calcium influx, suppressing presynaptic neurotransmitter release, and alleviating neuronal hyperexcitability (228, 229). Research from South Korean scientists revealed that intrathecal co-administration of pregabalin with P2X3/7 inhibitors and κ-opioid receptor agonists enhances analgesic efficacy in a combinatorial manner, suggesting that multi-targeted modulation of ion channels represents a promising strategy for pain management (230). Similarly, mirogabalin, which shares pregabalin’s mechanism of targeting the α2δ subunit but exhibits distinct ligand selectivity and slower dissociation kinetics, has demonstrated superior analgesic potency, enhanced safety profiles, and significantly reduced adverse effects in clinical trials compared to pregabalin (231). Additionally, synergistic analgesic effects have been observed when combining pregabalin with Kv7 potassium channel openers (flupirtine and retigabine), achieved through dual regulation of voltage-gated calcium and potassium channels. These findings underscore the potential of multi-mechanistic ion channel modulation as a refined approach for developing next-generation analgesics (232). Duloxetine, a serotonin-norepinephrine reuptake inhibitor, exerts its analgesic effects not only through inhibition of neurotransmitter reuptake but also via multimodal modulation of ion channels. It suppresses peripheral nociceptive signaling by blocking transient currents of Nav1.7 sodium channels (233) and further regulates TRP channel families, specifically inhibiting TRPC1/TRPC5 and TRPC4 channel activity to reduce calcium influx while decreasing current densities of TRPM2 and TRPV1 channels (234), thereby mitigating calcium overload-induced oxidative stress and apoptosis (235). Notably, although co-administration with minocycline improves mechanical allodynia by suppressing microglial activation, it exerts no significant alleviation of cold allodynia (236), underscoring the pathway-specific nature of duloxetine’s analgesic actions. A meta-analysis (237) demonstrated that 8% capsaicin effectively alleviates pain in patients with painful diabetic peripheral neuropathy, with fewer central adverse effects compared to oral analgesics. Capsaicin exerts its analgesic effects primarily by agonizing the TRPV1, triggering calcium influx that induces transient release followed by depletion of neuropeptides such as substance P from nerve terminals, thereby reducing nociceptive sensitization (238). Hagenacker et al (238). further revealed that capsaicin differentially modulates L-type, N-type, and T-type voltage-activated calcium channel currents in DRG neurons, with significant suppression of calcium channel activity in small-diameter neurons, potentially influencing neuronal excitability and neurotransmitter release. Additionally, capsaicin enhances intracellular sodium concentration ([Na⁺]i) in DRG neurons via TRPV1 activation, which indirectly suppresses voltage-gated sodium channel currents, attenuating neuronal excitability and action potential propagation. Intriguingly, high-concentration capsaicin selectively inhibits the sustained potassium current (IK), inducing hyperactivation of nociceptive neurons, which may contribute to its paradoxical hyperalgesic effects (239). These findings provide novel insights into the dual pro-nociceptive and analgesic mechanisms of capsaicin, highlighting its complex modulation of ion channel networks in pain pathways.

Regulating blood sugar levels stands as the fundamental approach in the management of DNP. GLP-1 receptor agonists, exemplified by semaglutide, have demonstrated significant efficacy in stimulating insulin secretion and reducing blood glucose levels effectively (240, 241). Semaglutide’s extended dosing interval enhances patient adherence to medication regimens, offering renewed optimism for the chronic management of diabetes. Presently, innovative diabetes treatments are under clinical investigation, including bimagrumab, orforglipron, and umbilical cord-derived mesenchymal stromal cells (242–244). These novel therapies are set to expand the horizons of diabetes care. Nonetheless, the proliferation of these treatments has intensified the focus on diabetes-related complications. In contrast to the diverse array of new hyperglycemia medications, DNP treatment options remain limited. Moreover, the effectiveness of current drugs can be variable among individuals and may entail certain side effects (245). Consequently, investigating the etiology of DNP, identifying novel therapeutic targets, and formulating new pharmaceuticals are crucial for easing patient symptoms and enhancing their quality of life. The application of herbal medicine, including traditional Chinese medicine, has a millennia-long history in disease treatment. Notable examples include drugs like artemisinin (246), arsenic trioxide (247) (a primary component of arsenic), and vincristine (248), which, following rigorous scientific validation, have been globally recognized for their potent effects against malaria and various cancers, including leukemia. Therefore, investigating the monomeric constituents and composite formulations within traditional Chinese medicines, in conjunction with contemporary pharmacological techniques, may offer novel insights and pathways for the research and development of DNP therapeutics.

The pathogenesis of diabetic neuropathic pain (DNP) involves complex interactions between neurons and glial cells (microglia, astrocytes, and Schwann cells), with dysregulation of ion channels playing a central role in abnormal nociceptive signaling. Neuronal hyperexcitability arises from imbalanced sodium (Nav1.7/1.8 overactivation), potassium (KCNQ functional suppression), and calcium channels (TRPV1/TRPM8-mediated calcium overload), while glial cells amplify pain through pro-inflammatory cytokine release, synaptic plasticity modulation, and neurotrophic dysregulation. Ion channel-targeted therapies (Nav1.8 inhibitors, Kv7 activators, TRP modulators) show promise by reducing neuronal excitability and glial inflammation. However, attention should be paid to the impact of gender differences on the function of ion channels. When developing new drugs targeting ion channels, gender-specific mechanisms should be incorporated to optimize clinical efficacy.
